# Older Adult Learners’ Contributions to Enhancing Age-Friendly Practices Within the Classroom

**DOI:** 10.1093/geroni/igaa057.1787

**Published:** 2020-12-16

**Authors:** Margaret Manoogian

**Affiliations:** Western Oregon University, Monmouth, Oregon, United States

## Abstract

Interest in college courses that focus on aging attract a variety of students. For older adults, the decision to take college courses may be fueled by a desire to engage in life-long learning, finish degrees that have been long desired, or retool and change careers. Older adults 65 years and older living in Oregon may audit courses for free in four-year public universities. Older auditors and degree-seeking students often work closely with faculty in providing support on course projects and sharing developmental insights that stimulate discussions about aging pathways. This presentation outlines the ways in which older adults contribute to educational conversations, mentor younger learners, and enhance classroom/university environments. How older adult learners have provided support in workshops, aided students in career decision-making, connected students to community resources, and provided contacts for intergenerational community activities will also be highlighted.

